# Detection of Bovine Brucellosis Antibodies in Serum and Milk Using Quantum Dot Microspheres Immunochromatographic Assay

**DOI:** 10.3390/microorganisms14051057

**Published:** 2026-05-08

**Authors:** Mingze Chen, Xin Yan, Jialu Zhao, Jingjing Xu, Mingjun Sun, Weixing Shao, Shufang Sun, Qiuming Du, Peipei Zhang, Shixiong Sun, Haobo Zhang, Mengda Liu, Xiangxiang Sun, Xiaoxu Fan, Wenlong Nan

**Affiliations:** 1 Laboratory of Zoonoses, China Animal Health and Epidemiology Center, Qingdao 266032, China; 18943320397@163.com (M.C.); yanxin@cahec.cn (X.Y.); sunmingjun@cahec.cn (M.S.); shaoweixing@cahec.cn (W.S.); sunshufang@cahec.cn (S.S.); zhangpeipei@cahec.cn (P.Z.); sunshixiong01@126.com (S.S.); zhanghaobo@cahec.cn (H.Z.); liumengda@cahec.cn (M.L.); sunxiangxiang@cahec.cn (X.S.); 2 College of Agriculture, Yanbian University, Yanji 133000, China; 3 Key Laboratory of Animal Biosafety Risk Prevention and Control (South), Ministry of Agriculture and Rural Affairs, Qingdao 266032, China; 4 College of Veterinary Medicine, Qingdao Agricultural University, Qingdao 266109, China; zjlu2156@163.com; 5 Animal Husbandry and Fishery Development Service Center, The 12th Division of Xinjiang Production and Construction Corps, Urumqi 830088, China; xjjhappygo@163.com; 6 Yanbian Animal Disease Prevention and Control Center, Yanji 133000, China; duqiuming007@163.com

**Keywords:** brucellosis, fluorescence microsphere immunochromatography, diagnosis

## Abstract

Brucellosis, a zoonotic disease caused by *Brucella*, requires rapid, accurate, and sensitive diagnostic methods for effective prevention and control. This study presents the development of a fluorescence microsphere immunochromatographic assay (QDMs-ICA) for detecting anti-Brucella antibodies in bovine serum and milk. Lipopolysaccharide (LPS) from the *Brucella abortus* strain A19 was immobilized on the nitrocellulose membrane (NC membrane) as the test line (T-line), while rabbit anti-SPG polyclonal antibody was applied as the control line (C-line). Recombinant streptococcal protein G conjugated with quantum dot microspheres (QDMs-SPG) served as the detection conjugate. After optimizing the preparation parameters of QDMs-ICA, the method demonstrated sensitivities of approximately 0.98 IU/mL for bovine serum and 1.56 IU/mL for milk. No cross-reactions were observed with antibody-positive sera from *Coxiella burnetii*, *Mycobacterium avium paratuberculosis*, *Mycobacterium tuberculosis*, *Chlamydia abortus*, *Bacillus anthracis*, *Escherichia coli* O157:H7, *Vibrio cholerae* or *Salmonella*, indicating excellent specificity. In intra- and inter-batch repeatability tests, the coefficient of variation (CV) remained below 15%, confirming good reproducibility. The detection limit remained stable after storage at 37 °C for 7 days. Parallel testing of 150 bovine serum samples and 80 milk samples showed a high degree of concordance with the ID-VET commercial kit, with coincidence rates of 97.3% and 96.3%, respectively. These results demonstrate that QDMs-ICA offers high specificity, sensitivity, repeatability, and reliability, making it an effective tool for the rapid detection and epidemiological monitoring of brucellosis.

## 1. Introduction

Brucellosis, caused by bacteria of the genus *Brucella*, is one of the most prevalent zoonotic diseases worldwide. The estimated global incidence of human brucellosis exceeds 500,000 cases annually. The disease also causes substantial economic losses exceeding billions of dollars in the livestock industry due to animal abortion, infertility, retained placenta, and orchitis. According to the World Organisation for Animal Health (WOAH), over 110,000 brucellosis outbreaks have been reported globally in the past five years. *Brucella abortus* accounts for more than 70% of these cases, making it the dominant pathogenic species in bovine brucellosis [1]. With the exception of certain countries in Western and Northern Europe, along with Canada, Japan, Australia, and New Zealand, which are considered brucellosis-free, the disease remains widespread, particularly in developing regions such as the Middle East, the Mediterranean, sub-Saharan Africa, and India [2]. Humans can contract brucellosis through the consumption of unpasteurized raw cow milk, and typical clinical symptoms include undulant fever, hyperhidrosis, and arthritis [3], posing a serious threat to human health.

Accurate and timely diagnosis is critical for controlling and preventing brucellosis. Serological testing is the most commonly used method for diagnosing suspected infections, conducting epidemic monitoring, and performing epidemiological studies [4]. Widely used serological techniques include the rose-bengal plate agglutination test (RBPT), complement fixation test (CFT), enzyme-linked immunosorbent assay (ELISA), and milk ring test (MRT). However, these methods are often hindered by limitations such as low sensitivity, complex procedures, and the requirement for laboratory settings. RBPT has a specificity of over 95% but a sensitivity of only 70–85%, leading to high false-negative rates in low-titer samples. CFT features high specificity yet is time-consuming (requiring 12–24 h) and prone to manual operation errors. ELISA offers sensitivity up to 90–98% but relies on professional equipment and trained personnel, making it unsuitable for on-site rapid detection. MRT is cost-effective for milk screening, but its sensitivity is reduced by 15–20% due to milk fat interference [5,6,7,8]. These limitations collectively restrict the field application of traditional methods, highlighting the urgent need for a rapid, sensitive, and user-friendly diagnostic tool. As a result, there has been a continuous effort to develop rapid, simple, sensitive, and specific diagnostic methods for brucellosis.

The immunochromatographic assay (ICA) is a technique that leverages the specific binding of antigens and antibodies combined with chromatography. ICA offers benefits such as ease of use, quick results, and visually interpretable outcomes. Colloidal gold immunochromatographic (GICA) test strips, developed based on this principle, are widely used for on-site detection of diseases such as brucellosis, avian leukemia, canine distemper, and foot-and-mouth disease at the grassroots level. The quantum dot microsphere (QDM)-based test strip, a more recent innovation, retains the advantages of GICA while enabling quantitative fluorescence analysis [9], addressing the subjectivity associated with visual interpretation in traditional GICA methods [10]. Lipopolysaccharide (LPS), a major component of the *Brucella* outer membrane, not only acts as a potent endotoxin but also exhibits strong immunogenic properties. Compared to proteins, antibodies induced by LPS have a higher peak and longer persistence, making LPS a preferred antigen in the development of *Brucella* antibody detection assays [11]. In this study, a QDMs-ICA was developed for detecting anti-*Brucella* antibodies in bovine serum and milk, utilizing QDMs as markers and LPS as the coated antigen.

## 2. Materials and Methods

### 2.1. Material Sources

The standard positive serum for bovine brucellosis (titer 4000 IU/mL) was obtained from the China Institute of Veterinary Drug Control. Clinical samples, including 150 bovine serum samples and 80 raw cow milk samples, were collected from large-scale dairy farms. A convenience sampling strategy was applied within the selected farms, targeting dairy cows aged 2–6 years. Positive sera for *Mycobacterium avium paratuberculosis*, *Mycobacterium tuberculosis*, *Chlamydia species*, *Coxiella burnetii*, *Bacillus anthracis*, *Escherichia coli* O157:H7, *Vibrio cholerae*, *Yersinia enterocolitica O:9* and *Salmonella* were prepared and collected in our laboratory. QDMs (1 μmol/mL) were procured from Beijing NanoKing Biotechnology Co., Ltd. Nitrocellulose (NC) membranes, glass fiber membranes, serum sample pads, absorbent papers, PVC backing cards, and rabbit anti-SPG polyclonal antibody (5 mg/mL) were sourced from Beijing Bonree Biotechnology Co., Ltd. (Beijing, China). Recombinant streptococcal protein G (SPG) (6 mg/mL) was purchased from Bersee Technology Co., Ltd. (Beijing, China). Fetal bovine serum (FBS) was obtained from Gibco (Beijing, China), and the handheld fluorescence analyzer was provided by Tianjin Biovest Biotechnology Co., Ltd. (Tianjin, China).

### 2.2. LPS Extraction

The *B. abortus* strain A19 was cultured in tryptic soy broth (TSB) until the exponential growth phase. The culture was then heat-inactivated at 80 °C for 2 h. Bacterial cells were collected by centrifugation, and LPS was extracted using a bacterial LPS extraction kit. The sample was loaded onto a 12.5% polyacrylamide gel for SDS-PAGE and analyzed by silver staining, following previously described methods [10]. The LPS was lyophilized, weighed, and stored at −80 °C. Prior to use, it was diluted to a concentration of 0.1 mg/mL with 0.02 M Tris solution for further applications.

### 2.3. Coupling of QDMs and SPG

For the preparation of conjugates, 0.2 mL of 1 μmol/L microspheres was diluted to 0.5 μmol/L with an equal volume of 20 mmol/L MES buffer (pH 6.5). Subsequently, 4 μL of 20 mg/mL EDC-HCl and 4 μL of 20 mg/mL NHS were added to the mixture, which was incubated at 37 °C for 15 min. The mixture was then centrifuged at 8000 rpm for 20 min, and the supernatant was discarded. The microspheres were resuspended in 0.2 mL of 10 mmol/L MES buffer (pH 6.5) to yield activated microspheres. Immediately afterward, 0.08 mg of SPG was added to the activated microspheres and incubated at 37 °C for 1 h. A blocking agent (80 μL) was then added, followed by a 30 min incubation. The conjugate was centrifuged, the supernatant was discarded, and the pellet was resuspended in 400 μL of 0.02 M Tris buffer (pH 8) and stored in the dark at 4 °C (final concentration 0.5 μmol/L).

### 2.4. Assembly of Test Strips

For the assembly of the test strip, the T-line of the NC membrane was dispensed with LPS, while the C-line was dispensed with rabbit anti-SPG polyclonal antibody. The PVC backing card was assembled with an absorbent pad, the NC membrane, and the conjugate pad (QDMs-SPG) in sequence. The assembled strips were cut into 3.5 mm wide test strips using a strip-cutting machine, placed in vacuum bags with desiccant, and stored in the dark at 4 °C.

### 2.5. Optimization of the Reaction System

To achieve optimal detection performance, five key parameters were optimized sequentially using an orthogonal experimental design, with the P/N ratio as the core evaluation index. The preliminary reaction conditions were set uniformly, and the optimal conditions were determined by the highest P/N value.

#### 2.5.1. Optimization of Conjugate Pad Coating Parameters

To further optimize the conjugate pad conditions, QDMs-SPG was diluted four-fold, six-fold, and eight-fold with the QDMs diluent and applied to the conjugate pad at 6 μL/cm, 8 μL/cm, and 10 μL/cm, respectively. All other conditions remained as described earlier. The coating concentrations and amounts for the conjugate pad were optimized using an orthogonal experimental design. The positive serum prepared in Section 2.5 and FBS were tested in parallel, with three repetitions performed for each condition. The optimal conjugate pad coating concentration and amount were determined based on the P/N ratio.

#### 2.5.2. Optimization of T-Line and C-Line Coating Concentrations

For the T-line and C-line coating conditions, LPS and rabbit anti-SPG polyclonal antibody were diluted with the membrane-coating solution. The T-line coating concentration was tested at two-fold, four-fold, six-fold, and eight-fold dilutions, while the C-line coating concentration was tested at four-fold, six-fold, eight-fold, and ten-fold dilutions. The different T-line and C-line coating concentrations were combined according to the orthogonal experiment method. The positive serum and FBS, as described previously, were detected in parallel, with three repetitions. The optimal coating concentrations for the T-line and C-line on the NC membrane were selected based on the P/N ratio of the test strip.

#### 2.5.3. Optimization of Sample Dilution Ratio

The positive serum and FBS prepared in Section 2.5 were diluted with the sample diluent at ratios of 1:4, 1:8, 1:16, and 1:32. All other conditions were maintained as described in the first paragraph of Section 2.5. Parallel tests were conducted three times for each dilution, and the optimal sample dilution was selected based on the P/N value of the test strip.

#### 2.5.4. Optimization of Sample Diluent Formula

Various reaction diluents were prepared, with their types and formulations detailed in Table 1. The sample diluents were used to dilute the positive serum and FBS from Section 2.5. Parallel tests were again performed three times for each group, and the optimal sample diluent was determined according to the P/N value of the test strip.

#### 2.5.5. Optimization of Immunochromatographic Reaction Time

Under optimal reaction conditions, the positive serum and FBS prepared in Section 2.5 were tested with the selected sample diluent. Immunochromatographic times of 10, 15, 20, 25, 30, and 35 min were evaluated. Each group was tested in triplicate, and the optimal immunochromatographic time was determined based on the P/N value of the test strip.

### 2.6. Qualitative Analysis

To establish the cut-off value for QDMs-ICA in Brucellosis detection, 16 positive and 24 negative bovine serum samples, confirmed by tube agglutination test (SAT) and RBPT [4], along with 15 positive and 25 negative spiked milk samples, were selected. These samples represented a range of antibody titers (high, medium, and low) to ensure data comprehensiveness. The test strips developed in this study were used to detect the samples, and P/N values were calculated. The cut-off value was determined through receiver operating characteristic (ROC) curve analysis using GraphPad Prism version 10.0.0, with the Youden index (sensitivity + specificity − 1) as the criterion to select the optimal critical value, ensuring both diagnostic sensitivity and specificity are maximized.

### 2.7. Performance Evaluation

#### 2.7.1. Analytical Sensitivity

The standard positive bovine serum for Brucellosis was diluted four-fold to 500 IU/mL with FBS and then serially diluted from 500 IU/mL to 0.49 IU/mL for further use. A 400 IU/mL standard positive milk sample for Brucellosis (spiked milk sample) was prepared by mixing the standard positive bovine serum with raw cow milk at a 1:9 ratio. This spiked milk sample was then serially diluted from 400 IU/mL to 0.391 IU/mL for subsequent use. The test strips developed in this study were employed to simultaneously detect bovine serum and milk samples at each dilution gradient, and the P/N values were calculated. The minimum dilution at which the test strips detected the bovine serum and milk samples as positive was recorded, and the analytical sensitivities were compared.

#### 2.7.2. Analytical Specificity

Positive bovine sera for *Coxiella burnetii*, *Mycobacterium avium paratuberculosis*, *Mycobacterium tuberculosis*, *Chlamydia abortus*, *Bacillus anthracis*, *Escherichia coli* O157:H7, *Vibrio cholerae*, *Yersinia enterocolitica O:9*, *Salmonella*, as well as positive serum and FBS prepared in Section 2.5, were selected. Each serum was detected in parallel three times using the test strips, and the P/N values from the three repetitions were averaged.

#### 2.7.3. Repeatability Detection

Intra-batch repeatability was assessed using three positive bovine serum samples from the serial dilutions in Section 3.1 (250 IU/mL, 31.25 IU/mL, and 0.98 IU/mL) and FBS, along with three positive milk samples (400 IU/mL, 25 IU/mL, and 1.5 IU/mL) and negative milk samples. Test strips from the same batch were used for parallel testing, and the P/N values were recorded. The coefficient of variation (CV = (SD/mean) × 100%) [12] was calculated. For inter-batch repeatability, the same samples were tested with strips from different batches in parallel three times, and the P/N values were recorded. The CV was calculated. IBM SPSS Statistics 27 software was used for repeated measures analysis of variance to assess the error range of the detection system. A CV of less than 15% indicated that the method was repeatable for brucellosis detection.

#### 2.7.4. Storage Stability Detection

For storage stability testing, the prepared QDMs-ICA test strips from the same batch were placed into a cassette, vacuum-sealed with desiccant, and stored at a constant temperature of 37 °C. On the 1st, 3rd, 5th, and 7th days, the test strips were used to detect the positive serum diluted to 0.98 IU/mL and FBS from the serial dilutions in Section 3.1. Each sample was tested in triplicate, and the P/N values were recorded to assess the strips’ storage stability.

#### 2.7.5. Clinical Sample Detection

For method comparison, the test strips prepared in this study and the serum I-ELISA (ID-VET) kit were used to detect 150 bovine serum samples. Similarly, the test strips and the milk I-ELISA (ID-VET) kit were used to detect 80 milk samples. IBM SPSS Statistics 27 and MedCalc software (MedCalc 20.0) were used for statistical analysis. Consistency between QDMs-ICA and commercial ELISA was evaluated using Kappa statistics with 95% confidence interval (95% CI). The difference in positive rates between the two methods was analyzed by McNemar’s χ^2^ test. The Kappa coefficient (κ) was used to assess consistency, with the following classification: <0.20 as extremely poor, 0.21–0.40 as fair, 0.41–0.60 as moderate, 0.61–0.80 as good, and 0.81–1.00 as excellent [13]. The coincidence rate between QDMs-ICA and the ELISA kit was also evaluated.

## 3. Results

### 3.1. Optimization Results of the Optimal Coating Concentration and Application Volume of the Conjugate Pad

**Figure 1:**
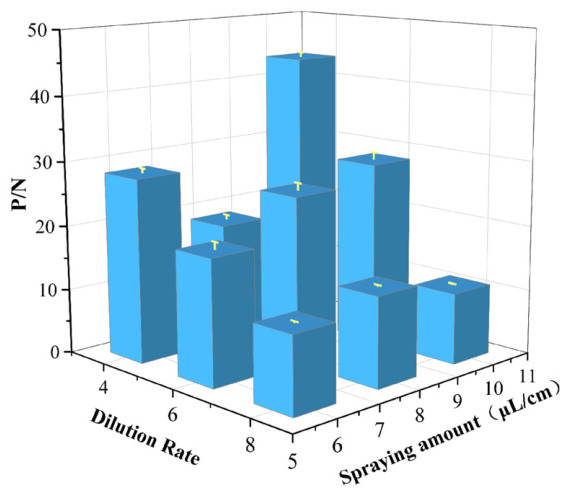
Optimization of conjugate pad coating concentration and application volume. P/N, ratio of positive sample reading to negative sample reading.

Data (Figure 1) demonstrated that the maximum P/N ratio (43.5) was obtained when QDMs-SPG was diluted 4-fold and coated at 10 μL/cm, which was determined as the optimal parameter for the conjugate pad.

### 3.2. Optimization Results of the Optimal Coating Concentrations of the T-Line and C-Line

**Figure 2:**
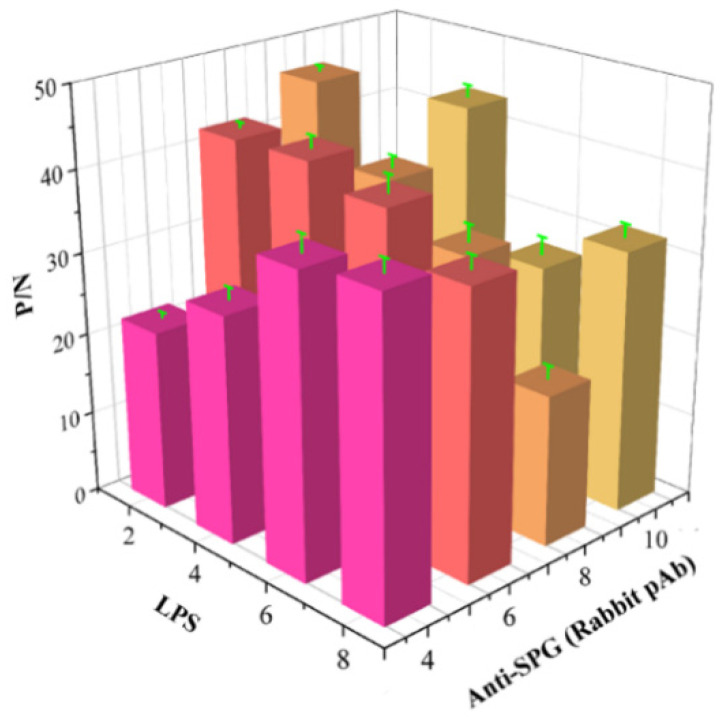
Optimization of coating concentrations on the T-line and C-line. P/N, ratio of positive sample reading to negative sample reading.

The maximum P/N ratio (46.62) was observed (Figure 2) when the T-line LPS was diluted 2-fold, and the C-line rabbit anti-SPG pAb was diluted 8-fold, which were selected as the optimal coating concentrations.

### 3.3. Sequential Optimization of Sample Dilution, Diluent Formulation, and Reaction Time

**Figure 3:**
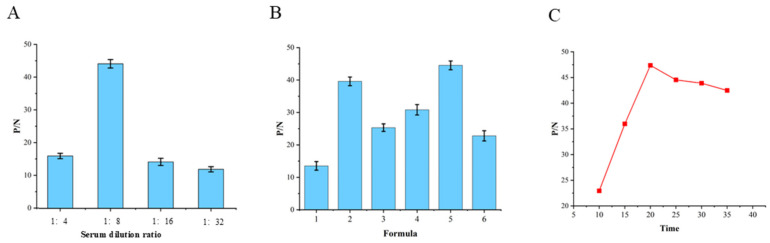
Optimization of sample dilution (**A**), sample diluent (**B**), and immunochromatographic time (**C**). P/N, ratio of positive sample reading to negative sample reading.

The optimal sample dilution ratio was 1:8, the optimal diluent was Formula 5, and the optimal immunochromatographic time was 20 min, as validated by the highest P/N values in sequential tests (Figure 3).

### 3.4. Determination of the Cut-Off Value

**Figure 4:**
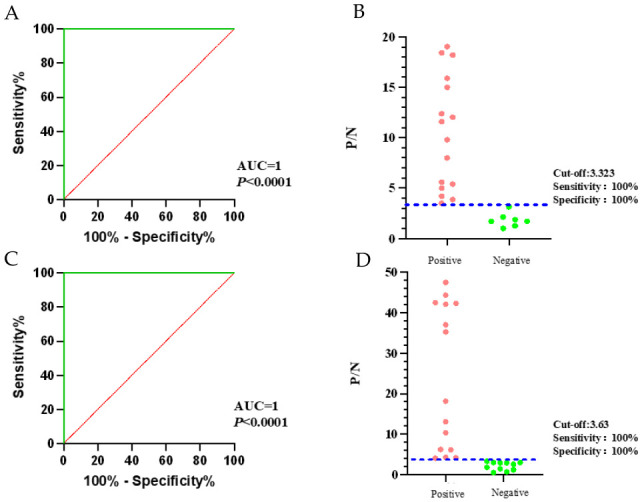
Determination of the cut-off value for Brucella antibodies. P/N, ratio of positive sample reading to negative sample reading.

The detected data were entered into GraphPad Prism version 10.0.0, and ROC curve analysis was performed to determine the cut-off value and generate an interactive scatter plot. For the detection of serum antibodies against bovine brucellosis, the results showed that at a cut-off value of 3.323, both diagnostic sensitivity and specificity were 100% (Figure 4A,B). For the detection of milk antibodies against bovine brucellosis, the results showed that at a cut-off value of 3.63, diagnostic sensitivity and specificity were again 100% (Figure 4C,D). The area under the curve (AUC) for both bovine serum and milk brucellosis detection was 1, with *p* < 0.0001, indicating the QDMs-ICA method possesses high diagnostic accuracy for bovine brucellosis antibody detection [14]. These results validate that the QDMs-ICA method is both accurate and reliable for brucellosis detection. It should be noted that it may be influenced by the limited sample size used for cut-off determination. External validation with larger, independent cohorts is recommended to further confirm these estimates.

### 3.5. Results of Analytical Sensitivity

Data (Table 2) demonstrated that the detection limit of this test strip for bovine serum samples was 0.98 IU/mL, while for milk samples, it was 1.56 IU/mL. The data is significantly lower than the 25 IU/mL (serum) reported by Li et al. [9] and 7.81 IU/mL (serum) reported by Kong et al. [15], showing a substantial improvement in detection sensitivity.

### 3.6. Results of Analytical Specificity

The data (Table 3) indicate that the test strip developed in this study did not cross-react with positive sera from *Coxiella burnetii*, *Mycobacterium avium paratuberculosis*, *Mycobacterium tuberculosis*, *Chlamydia species*, *Bacillus anthracis*, *Escherichia coli* O157:H7, *Vibrio cholerae* and *Salmonella*, thereby demonstrating excellent specificity. The only observed cross-reaction occurred with *Yersinia enterocolitica O:9*, which is attributable to the high similarity of antigenic epitopes between this strain and *Brucella*, making high-specificity differentiation inherently difficult.

### 3.7. Results of Repeatability Detection

Data (Table 4) demonstrated that for both intra-batch and inter-batch repeatability tests, the CVs for the three bovine serum samples with different titers were 3.96%, 5.76%, and 5.47%, and 1.99%, 6.95%, and 2.70%, respectively. The CVs for the three milk samples with different titers were 2.85%, 2.58%, and 4.28%, and 2.70%, 3.47%, and 4.76%, respectively. All CV values were below 15%, confirming that the test strip exhibits good repeatability.

### 3.8. Results of Storage Stability Detection

The data (Table 5) indicate that for test strips stored at 37 °C for 1, 3, 5, and 7 days, the P/N values of the 0.98 IU serum samples from Section 3.1 remained around 4, and no significant decrease in the minimum detection limit was observed. These results demonstrate that the QDM immunochromatographic test strips developed in this study exhibit good storage stability.

### 3.9. Results of Clinical Sample Detection

Data (Table 6) demonstrated that the Kappa consistency coefficients for bovine serum and milk samples were 0.871 (95% CI: 0.782–0.935) and 0.900 (95% CI: 0.806–0.955), respectively, both greater than 0.8, indicating excellent agreement. McNemar test showed no significant difference between the two methods (serum: χ^2^ = 0.625, *p* = 0.429; milk: χ^2^ = 0.500, *p* = 0.480). The total coincidence rates were 97.3% and 96.3%, respectively. The result indicates excellent agreement between QDMs-ICA and the commercial ELISA kit, which meets the clinical application requirements for bovine brucellosis serological screening and epidemiological monitoring.

## 4. Discussion

Serological detection is essential for diagnosing brucellosis in both humans and animals. While pathogen isolation and nucleic acid detection are valuable, serological diagnostic tests are preferred in most endemic regions due to their simplicity and applicability in resource-limited clinical settings [16,17]. Despite this, the widely recommended serological methods by WOAH have limitations. The RBT is commonly used for preliminary screening of brucellosis due to its high specificity, but it suffers from relatively low sensitivity [5]. The SAT, widely employed to assess vaccine efficacy in ruminants, is hindered by cumbersome procedures, time consumption, low sensitivity, and susceptibility to subjective interpretation bias [6]. While ELISA and FPA methods offer good sensitivity and specificity, their high reagent costs and requirement for specialized technicians limit their widespread use [7]. Although the MRT is cost-effective and easy to operate, its sensitivity can be compromised by factors such as the fat content of dairy products [8]. According to national standards for Brucella diagnostics in China, ICA has been recognized as a suitable method for detecting disease-free livestock herds, implementing eradication programs, and monitoring population prevalence. Unlike traditional GICA, the novel QDMs-ICA, when paired with a portable fluorescence reader, provides digital T and C values within 10–20 min, offering enhanced sensitivity and reduced background interference.

Compared with previously reported QDM-based immunochromatographic assays for brucellosis, the method developed in this study exhibits distinct advantages in antigen selection, detection matrix, and sensitivity. For instance, the QDMs test strip developed by Li et al. [9] using Brucella whole-cell proteins achieved a 96.98% coincidence rate with the SAT in detecting 199 serum samples, but its minimum detection limit was only 25 IU/mL. Similarly, Kong et al. [15] prepared QDMs test strips using Brucella outer-membrane proteins OMP22 and OMP28, which showed a 97.3% coincidence rate with the RBPT in detecting 150 serum samples, with a minimum detection limit of 7.81 IU/mL. LPS, the most crucial antigen during both infection and vaccination, induces antibodies with a high peak and prolonged duration, making it the most commonly used antigen in Brucella antibody detection technologies [11,18]. While whole-cell proteins encompass all bacterial protein components and theoretically improve detection sensitivity, their complex composition reduces specificity and complicates batch-to-batch quality control. ICA technology based on recombinant outer-membrane proteins offers relatively better specificity, but the antibodies induced have a lower peak and shorter duration, resulting in overall sensitivity inferior to that of LPS [19]. In contrast, the QDMs test strip based on LPS developed in this study demonstrates a significantly lower minimum detection limit of 0.98 IU/mL, which is much lower than those reported by Li and Kong et al. [9,15]. Compared with the QDMs-ICA based on whole-cell proteins (Li et al. [9]) and outer-membrane proteins (Kong et al. [15]), our LPS-based QDMs-ICA reduces the serum detection limit from 7.81 to 25 IU/mL to 0.98 IU/mL, improving sensitivity to 87.6–96.1%. Furthermore, this test strip extends the detection matrix’s application range, enabling simultaneous detection of milk samples, with a minimum detection limit of 1.56 IU/mL. Detecting Brucella antibodies in milk samples offers advantages by avoiding the impact of blood collection on milk production in dairy cows, reducing the infection risk for veterinarians and related personnel, and facilitating bulk tank milk screening in intensive farms. Additionally, the test strip’s performance remains unaffected after raw milk is treated at 72 °C for 15 min, indicating that it can also be used for detecting pasteurized milk. This makes the test strip applicable across the entire supply chain, from raw farm milk to pasteurized commercial milk, which holds significant public health value in preventing the transmission of foodborne brucellosis and ensuring dairy safety. Based on its principle-based universality, this study also evaluated the detection performance of sheep serum, but the results fell short of expectations, with a sensitivity below 85%. The next step involves system optimization to enhance the method’s applicability across species. The development of this method for milk sample testing facilitates real-time, high-frequency monitoring of brucellosis status in livestock herds or lactating animals through non-invasive and convenient approaches, thereby supporting the formulation of targeted prevention and control strategies. Next, this study will further validate the method’s performance in practical applications such as ranches by employing larger-scale sample cohorts covering diverse geographical regions, thereby reducing the sampling bias observed in the current research. In summary, this detection method remains a highly promising field screening tool due to its rapid testing capability, user-friendly operation, and dual applicability to both serum and milk samples. This study evaluated the QDMs-ICA test strip for diagnosing brucellosis, showing 97.3% and 96.3% coincidence rates with the ID-VET ELISA commercial kit for bovine serum and milk, respectively. The test strip demonstrated good sensitivity, specificity, and repeatability. In conclusion, the test strip developed in this study is simple, rapid, sensitive, and specific, making it suitable for on-site screening of brucellosis and a valuable tool for brucellosis prevention, control, and dairy safety monitoring.

## Figures and Tables

**Table 1:** Reaction diluents with different formulations.

Type	Preparation Method
Formula 1	Water
Formula 2	0.02 M/mL PB, pH = 7.4
Formula 3	0.02 M/mL PB, pH = 7.4 + 0.1% tween 20
Formula 4	0.02 M/mL PB, pH = 7.4 + 1% NaCl + 0.1% tween 20
Formula 5	0.02 M/mL PB, pH = 7.4 + 1% casein + 0.1% tween 20
Formula 6	0.02 M/mL PB, pH = 7.4 + 1% skim milk powder + 0.1% tween 20

**Table 2:** Sensitivity detection of QDMs-ICA for bovine serum and milk.

Serum Antibody Titer (IU/mL)	ICA Test Result	Judgment	Milk Antibody Titer (IU/mL)	ICA Test Result	Judgment
500	48.53 ± 2.61	Positive	400	36.84 ± 1.05	Positive
250	53.79 ± 2.13	Positive	200	36.18 ± 2.16	Positive
125	50.34 ± 3.89	Positive	100	31.76 ± 3.45	Positive
62.5	49.33 ± 4.93	Positive	50	29.58 ± 1.97	Positive
31.25	41.65 ± 2.40	Positive	25	20.52 ± 0.53	Positive
15.63	26.91 ± 2.63	Positive	12.5	19.09 ± 2.16	Positive
7.81	20.35 ± 2.67	Positive	6.25	10.91 ± 1.56	Positive
3.9	10.00 ± 1.01	Positive	3.13	8.53 ± 0.90	Positive
1.95	7.57 ± 1.02	Positive	1.56	4.21 ± 0.18	Positive
0.98	3.84 ± 0.21	Positive	0.781	2.45 ± 0.25	Negative
0.49	2.49 ± 0.12	Negative	0.39	1.56 ± 0.14	Negative
Negative	1	Negative	1	1	Negative
Control					

**Table 3:** Results of the specificity evaluation of Brucella antibodies.

Antibody-Positive Serum	P/N Value	Judgment
*Coxiella burnetii*	1.64 ± 0.65	Negative
*Mycobacterium avium paratuberculosis*	0.98 ± 0.24	Negative
*Mycobacterium tuberculosis*	0.63 ± 0.11	Negative
*Chlamydia abortus*	0.88 ± 0.30	Negative
*Bacillus anthracis*	0.76 ± 0.02	Negative
*Escherichia coli O157:H7*	1.03 ± 0.08	Negative
*Vibrio cholerae*	0.72 ± 0.15	Negative
*Salmonella*	0.95 ± 0.05	Negative
FBS	1	Negative
*Brucella*	46.44 ± 1.73	Positive

Note: P/N, ratio of positive sample reading to negative sample reading.

**Table 4:** Analytical Repeatability Test of QDMs-ICA.

Titer (IU/mL)	Intra-Batch Coefficient of Variation		Inter-Batch Coefficient of Variation	
	Mean ± SD	CV (%)	Mean ± SD	CV (%)
250 *^a^*	53.79 ± 2.13	3.96	52.84 ± 1.05	1.99
31.25 *^a^*	41.65 ± 2.40	5.76	45.47 ± 3.16	6.95
0.98 *^a^*	3.84 ± 0.21	5.47	3.90 ± 0.105	2.70
400 *^b^*	36.84 ± 1.05	2.85	38.95 ± 1.05	2.70
25 *^b^*	20.52 ± 0.53	2.58	21.26 ± 0.74	3.47
1.56 *^b^*	4.21 ± 0.18	4.28	4.42 ± 0.21	4.76

Note: *^a^*: Bovine serum, *^b^*: Milk, CV, coefficient of variation, SD, standard deviation.

**Table 5:** Evaluation of the storage stability of QDMs-ICA.

Days of Storage at 37 °C (d)	Mean Value
1	4.10 ± 0.14
3	4.45 ± 0.28
5	4.68 ± 0.02
7	3.85 ± 0.14

**Table 6:** Detection of clinical samples.

	Clinical Samples		QDMs-ICA Method		
			Positive	Negative	Total
ELISA (ID-VET)	Bovine Serum	Positive	90	6	96
		Negative	3	51	54
		Total	93	57	150
		Kappa value		0.871	
	milk	Positive	19	1	20
		Negative	2	58	60
		Total	21	59	80
		Kappa value		0.90	

## Data Availability

The original contributions presented in the study are included in the article. Further inquiries can be directed to the corresponding authors.
